# Wastewater Biosolid Composting Optimization Based on UV-VNIR Spectroscopy Monitoring

**DOI:** 10.3390/s16111919

**Published:** 2016-11-15

**Authors:** Beatriz Temporal-Lara, Ignacio Melendez-Pastor, Ignacio Gómez, Jose Navarro-Pedreño

**Affiliations:** Department of Agrochemistry and Environment, University Miguel Hernández of Elche (UMH), Avenida Universidad s/n Edificio Alcudia, 03202 Elche, Spain; btemporal@umh.es (B.T.-L.); imelendez@umh.es (I.M.-P.); jonavar@umh.es (J.N.-P.)

**Keywords:** wastewater sludge, biosolid compost, spectroscopy, PLS

## Abstract

Conventional wastewater treatment generates large amounts of organic matter–rich sludge that requires adequate treatment to avoid public health and environmental problems. The mixture of wastewater sludge and some bulking agents produces a biosolid to be composted at adequate composting facilities. The composting process is chemically and microbiologically complex and requires an adequate aeration of the biosolid (e.g., with a turner machine) for proper maturation of the compost. Adequate (near) real-time monitoring of the compost maturity process is highly difficult and the operation of composting facilities is not as automatized as other industrial processes. Spectroscopic analysis of compost samples has been successfully employed for compost maturity assessment but the preparation of the solid compost samples is difficult and time-consuming. This manuscript presents a methodology based on a combination of a less time-consuming compost sample preparation and ultraviolet, visible and short-wave near-infrared spectroscopy. Spectroscopic measurements were performed with liquid compost extract instead of solid compost samples. Partial least square (PLS) models were developed to quantify chemical fractions commonly employed for compost maturity assessment. Effective regression models were obtained for total organic matter (residual predictive deviation—RPD = 2.68), humification ratio (RPD = 2.23), total exchangeable carbon (RPD = 2.07) and total organic carbon (RPD = 1.66) with a modular and cost-effective visible and near infrared (VNIR) spectroradiometer. This combination of a less time-consuming compost sample preparation with a versatile sensor system provides an easy-to-implement, efficient and cost-effective protocol for compost maturity assessment and near-real-time monitoring.

## 1. Introduction

The treatment of domestic and industrial wastewater is usually associated with the production of an organic matter–rich (and potentially dangerous due to the presence of pathogens, and even heavy metals and micropollutants) sludge [1,2]. The implementation of new environmental regulations is promoting an increase in the quantities of wastewater sewage sludge due to the constant rise in the number of households connected to sewers and the increase in the level of treatment. As an example, the production of sewage sludge in the European Union increased from 5.5 million tons of dry matter in 1992 [3] to more than 10 million tons in 2012 (data calculated from [4]). It represents an average per capita sewage sludge dry matter production of 19.0 kg/inhabitant for the European Union in 2012, with a maximum of 58.9 kg/inhabitant for Spain (data calculated from [4]). That huge amount of wastewater sludge requires adequate analysis and treatments to ensure its reuse. Options for sludge treatment include stabilization (e.g., composting, anaerobic digestion), thickening, dewatering, drying and incineration [5].

Composting is a stabilization process of organic wastes, such as wastewater sludge, based on the microbiological decomposition of the organic matter to produce compost (humus) [5,6,7]. The composting process involves the complex destruction of organic matter coupled with the production of humic acids (HA) to produce a stabilized end product [1]. The correct development of the composting process, from the raw organic material to a mature compost end product, is associated with optimum conditions, such as moisture, the carbon-nitrogen ratio or temperature [1,5]. The complexity of the process and the large number of control variables (physical, chemical and microbiological) has promoted the exploration of new methods and techniques for proper monitoring of the composting process. Diverse techniques had been proposed for monitoring the composting processes such as the employment of microbiological respiration indices [8], chemical fractionation [9], or spectroscopic analyses [10] at several wavelength ranges. The last two techniques can be coupled for a better assessment of compost maturity. For example, the progressive formation of humic-like substances during the composting process is an appropriate way to assess the compost evolution [11]. Moreover, the visible-ultraviolet (Vis-UV) spectrum measured at different compost liquid extracts can be used to predict the humification degree [12]. Consequently, the combination of both techniques is a suitable way for monitoring the composting process.

The continuous monitoring of chemical parameters in wastewater treatment processes is a subject of major concern for the water industry [13,14,15,16]. It should allow a better control of the treatment process for compliance with environmental regulations at the lowest cost and energy consumption. However, many traditional parameters employed for wastewater treatment control (e.g., biochemical oxygen demand, chemical oxygen demand) or biosolid composting assessment (e.g., total carbon, total nitrogen) are time-consuming and/or require equipment with an unviable cost for the laboratory control of many wastewater treatment plants (WWTP), requiring the shipment of samples to specialized laboratories. It hinders the (near) real-time control of relevant chemical parameters of wastewater and biosolid compost. Additionally, the hostile environment in which sensors have to be located hampers their implementation [17]. However, the idea of employing optical-electronic sensors for online monitoring of wastewater treatment processes is not recent [18], but it is generating increasing attention [13,14,16,17,19,20]. They are a suitable method for fast data acquisition, which limits direct contact of the sensor with the wastewater or compost, and provide a quantitative assessment of chemical compounds through previous calibration experiments. Previous research has suggested the employment of ultraviolet [21], ultraviolet-visible [22], visible–near-infrared (VNIR) [23,24], or ultraviolet and visible-–near-infrared [25] spectroscopy for monitoring wastewater sludge compost. However, compost samples may require a complex and time-consuming preparation (e.g., compost drying and sieving) before their spectroscopic analyses in order to minimize the physical-chemical heterogeneity and different humidity contents of the samples. Both sources of uncertainty restrict the (near) real-time monitoring of the compost process. It is therefore interesting to perform a compost sample preparation prior to spectroscopic analysis, which minimizes both uncertainties by obtaining liquid compost extracts that can be analyzed (chemically and spectroscopically) and related with different characteristics of the composting degree. In fact, a combination of a faster and simpler compost extraction method and a robust and cost-effective optical-electronic sensor would be desired for a wider implementation of spectroscopy systems in (near) real-time monitoring of the composting process.

The aim of this study was the establishment of a feasible methodology to improve the composting process’s control based on quantifying its degree of maturity with UV-VNIR spectroscopy of liquid compost extracts. The objective of this manuscript was the assessment of the best spectral ranges to quantify compost maturity based on regression models among compost chemical parameters and humification indices, and the UV-VNIR spectra obtained for the liquid compost extracts. This purpose could be satisfied by combining our previously developed one-step wastewater biosolid compost extraction method for chemical fractioning [26] and relatively simple and cost-effective spectroscopy systems.

## 2. Material and Methods

Wastewater biosolid compost was processed in a composting tunnel located within a municipal WWTP at the Alicante province (Spain). This facility includes four partially closed composting tunnels (63 m length, 3 m width and 1.75 m height). The compost was obtained by processing a mixture of municipal sewage sludge, sawdust and chopped wheat straw for a 4:3:1 proportion, respectively. The composting process is improved by adding supplementary waste materials (e.g., wheat straw, rice straw, cotton waste or sawdust) as bulking agents. They permit adequate gas exchange, absorbs excess moisture, and prevent excessive compaction of the composting substrate by providing the structural support to create interparticle voids [27]. Both bulking agents provided carbon to the wastewater sludge to optimize the carbon:nitrogen (C/N) ratio. The mixture was automatically turned three times per week with a mechanical turner (Volteco, model 2030, Ponzano Veneto, Italy), making the advancement of compost along the tunnels (compost advancement speed between 0.6 and 1.2 m/min; turning speed of the drum between 70 and 100 rpm). Finally, compost was moved to an outdoor pile for completing the maturation process.

### 2.1. Compost Sampling and Analytical Methods

Sampling points were located at different stages of a sewage sludge composting process. The samples differed in their degree of maturity by their different period of residence. Compost samples were obtained at seven composting stages: 14, 21, 28, 35, 42, and 49 days of composting process within the tunnel and an additional one at the outdoor compost pile (57 days of compost maturity). For each sample point, four subsamples representing the width of the composting tunnel were selected; each one was taken at a randomly chosen depth. A total of eighty four independent compost samples (*n* = 84) were obtained. Independent compost samples were obtained at different depths and analyzed by triplicate. Compost samples were immediately stored under cold conditions (~4 °C) to minimize the alteration of the samples. All chemical analyses were done within hours from the sampling. Further details of the sampling procedure can be found at Temporal-Lara et al. [26].

We analyzed organic matter related chemical parameters and commonly proposed compost extracts chemical fractions to quantify its maturity [26,28,29]. Total organic matter content (TOM) was determined by loss on ignition at 450 °C for 6 h (UNE-EN 13.039 2001). Total carbon (TC) was determined by dry combustion using an Elemental Analyzer (TruSpec). Total extractable carbon (TEC) was obtained with sodium pyrophosphate 0.1 M (1:10 *w*:*v*) according to the one-step extraction procedure of Temporal-Lara et al. [26]. An aliquot of TEC extracts was acidified to pH below 1 and allowed to stand at room temperature overnight. Soluble fulvic acids (FA) and other non-humic substances were then separated from the precipitated humic acids (HA), determining the organic carbon in the supernatant according to Swift [30]. The HA was calculated by the difference between TEC and FA. Nitrogen content was determined with the modified total Kjeldahl nitrogen (TKN) method (UNE 77318:2001). Several compost maturity ratios were also computed, namely: C/N, the humification ratio (HR = TEC/TOC) and humification index (HI = HA/TOC) [31]. Compost extracts were immediately stored under cold conditions for spectroscopic analysis.

### 2.2. Spectral Measurements

Compost extracts were analyzed with two different spectroscopic systems to obtain UV and VNIR spectra. UV measurements were performed with a Unicam UV-500 double-beam spectrophotometer (Thermo Scientific, Waltham, MA, USA). It covers the wavelength range 190–900 nm, with an accuracy of ±0.3 nm in the spectral range 250–500 nm and ±0.5 nm from 190 to 250 nm and 500 to 900 nm. Compost extracts were manually placed in the default 10 mm cuvette holder. The spectrophotometer was controlled with a computer for scanning absorbance spectra in the spectral range 220–400 nm. A changeover between deuterium and tungsten lamps was setup at 325 nm. Spectra were acquired with a 1 nm data interval and a scan speed range of 120–1200 nm/min that provide adequate data for UV scans.

VNIR measurements were performed with an Analytical Spectral Devices (ASD) Field Spec Hand Held VNIR radiometer (ASD Inc., Boulder, CO, USA). It covers the wavelength range of 325–1075 nm, which approximates with the visible and short wave near infrared (SW-NIR) spectral regions, with an accuracy of ±1 nm and a resolution of <3 nm at 700 nm. The radiometer was connected through a fiber optic cable to an Ocean Optics (Leesburg, FL, USA) 10 mm cuvette holder where compost extracts were placed. Another fiber optic cable was connected from the cuvette holder to an ASD Fiber Optic Illuminator^®^ as light source. This systems enables the illumination of the cuvette from one of its faces and the transmittance record from the opposite cuvette face. Five radiometric measurements (with 15 automatic replicate spectra per measurement) were taken for each sample. The dark current (detector background) and reference spectra were taken immediately before each spectral measurement. The five radiometric measurements were visually inspected and then averaged to obtain a single spectrum per sample.

Random noise of the spectra was minimized by applying a Savitzky-Golay algorithm across a moving window of 10 nm with a third-order polynomial [32]. All compost extracts spectral measurements were done in quartz glass cuvettes using the compost extractant (sodium pyrophosphate 0.1 M with a proportion 1:10 *w*:*v*) as blank. Excessive turbidity was avoided by dilution of the liquid compost extracts (with sodium pyrophosphate 0.1 M with a proportion 1:5 *v*:*v*) before taking the spectral measurements in the cuvettes. Spectra were acquired at room temperature (24 ± 1 °C) within hours from the compost extraction.

### 2.3. Spectroscopic Analyses

Summary statistics of the chemical fractions obtained from the wastewater biosolid compost extracts were computed. Minimum, maximum, mean, standard deviation (St. Dev.) and coefficient of variation (CV) were included. Also, a comparison of the means through the normalized analysis of variance (ANOVA) was computed. We considered the position along the tunnel as the factor for a one-way ANOVA. Previously, the normal distribution and homogeneity of the variances were verified with the Kolmogorov-Smirnov and the Levene’s test, respectively.

Partial Least Squares Regression (PLSR) was employed to relate the chemical fractions of the compost extracts with the UV and VNIR spectra. PLSR has been designed to confront the situation that there are many, possibly correlated, predictor variables, and relatively few samples [33]. PLSR is a feasible quantitative multivariate modeling method for chemometrics [34] where highly detailed spectra data (i.e., high spectral resolution or number of bands) are employed to quantitatively predict a limited number of problem samples. PLSR models were developed for the UV (220–400 nm) and the VNIR (400–1000 nm) spectral ranges. Two additional models were developed for subsets of the VNIR spectra. These spectral subsets were identified with the visible (400–700 nm) and the near infrared (700–1000 nm) spectral ranges [35]. The selection and evaluation process of the PLSR models [33] was based on the following methodological procedure [33,36]: (1) the original 84 samples dataset was stratified randomly divided into a subset for model cross-validation (75% of the samples), and the remaining samples for independent test; (2) a leave-one-out (LOO) cross-validation (CV) procedure was used for the development of PLSR models; and (3) selected models were tested with the independent validation dataset in order to assess the predictive capabilities of the selected PLSR models.

Several diagnostic statistics were employed for PLSR models assessment. The root mean squared error (RMSE) was the main statistical parameter used to guide the number of model components or latent variables (LV) selection The number of optimal components and the selection of the better models were determined based on the lowest RMSE values for the adjusted CV with the LOO procedure (i.e., RMSECV). Cross-validation Pearson correlation coefficient (R^2^) was also applied as an illustrative diagnostic statistic. RMSE of the prediction dataset (RMSEP) was also computed for selected models evaluation. In addition, the residual predictive deviation (RPD) was used to determine the practical utility of the models in the prediction test stage. The models and predictions were classified according to Chang et al. [37] as successful (RPD > 2), moderately successful (RPD = 1.4–2) or not successful (RPD < 1.4). PLSR models and further statistical analyses were developed with the R statistical programming language [38]. PLS package [33] was employed for the development of PLSR models.

## 3. Results

**Figure 1:**
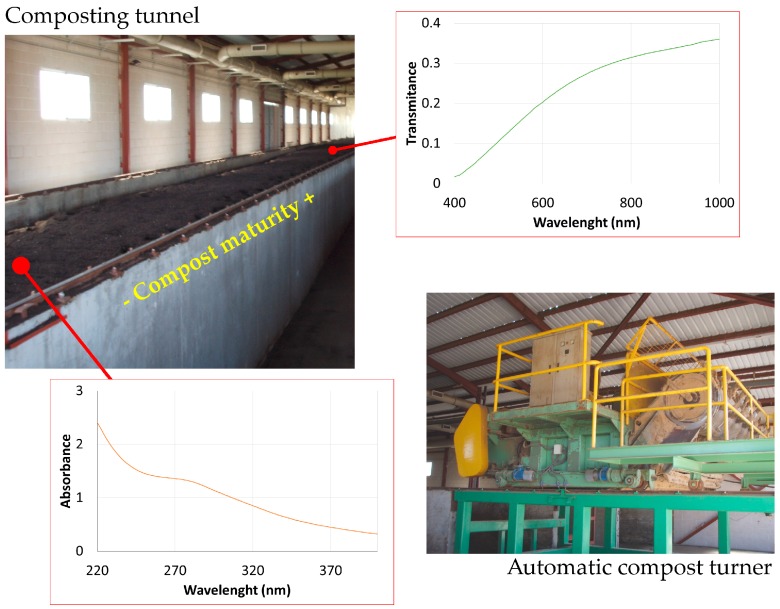
Composting tunnel and automatic compost turner. The figure composition includes characteristic UV and VNIR spectra of the samples collected at the beginning and the end of the composting process, respectively.

The compost samples collected at different locations within the composting tunnel exhibited different spectral patterns and chemical fractioning. Figure 1 shows an overview of the composting tunnel and a detailed image of the system employed for the aeration and mixture of the compost. We include an example of a characteristic absorbance UV spectrum obtained for samples located at the beginning of the composting process (14 days of maturity). A characteristic transmittance VNIR spectrum for a higher degree of compost maturity (49 days of maturity) was included as well. Both characteristic spectra do not exhibit relevant absorption or reflection bands that could be enhanced with a derivative analysis.

A statistical summary of the chemical parameters obtained from the wastewater biosolid compost extracts is shown in Table 1. Mean TOM and TOC were 70.8% and 38.6%, respectively, while the coefficient of variation was moderate (CV < 4.3%) for both parameters. We employed the ANOVA to reveal variations of the chemical parameters in the composting processes. In this case, significant differences (*p*-value < 0.001) were obtained for both chemical parameters. The mean TEC content of the samples was 653 mg/L, with a maximum of 903.2 mg/L. Average FA and HA concentrations were 237.5 mg/L and 415.4 mg/L, respectively. The coefficients of variation for both variables were very similar and slightly higher than for the TEC. Higher values of TEC, FA and HA were obtained in the first half of the tunnel. Significant differences (*p*-value < 0.05 for HA and *p*-value < 0.001 for TEC and FA) were obtained for the extractable chemical fractions. Non-significant differences were obtained for both the TKN and C/N ratio. The mean value of TKN was 3.0% while the maximum and minimum C/N values were 15.5% and 10.9%, respectively. Finally, the humification ratios revealed significant differences in the composting process (*p*-value < 0.001 for HR and *p*-value < 0.05 for HI). Mean values of the HR and HI were 17.0% and 10.8%, respectively.

PLSR models of the compost extracts’ chemical fractions were developed for different wavelength ranges (Table 2). The initial process of cross-validation was performed for all spectral ranges while the prediction test phase was applied for the better models of the previous stage. The selection of the better models was based on lower RMSE values. The number of latent variables or model components was selected based on the minimization of the RMSECV. The Pearson correlation coefficient (R^2^) was also computed for a general overview of the models’ performance.

Total organic matter was better quantified for the 700–1000 nm spectral range. The cross-validation test for the NIR spectra showed a RMSECV of 2.04 (i.e., 19.89%) and a medium-high Pearson correlation coefficient (R^2^ = 0.628). A RMSEP of 1.11 (i.e., 12.14%) and a RPD of 2.68 were obtained for the independent prediction test. The selected spectral range for the total organic carbon quantification was the NIR (700–1000 nm) as well. The Pearson correlation coefficient (R^2^ = 0.607) of the cross-validation was moderate. The RMESP was 26.60% and the RPD value revealed a moderately successful model (RPD = 1.66). The total extractable carbon exhibited a better correlation with VNIR spectra and especially with the visible spectral range. RMSECV was almost identical for the full VNIR (400–1000) and the visible (400–700) spectral ranges. However, the full VNIR spectral range was selected as the better model, which achieved the highest Pearson correlation coefficient (R^2^ = 0.810). The VNIR spectra were employed for the prediction test. The results of the RMESP (65.02% and 14.14%) were slightly higher but comparable with the RMSECV (57.08% and 12.41%) values, while the RPD was 2.07 for that model. The RMSECV values for the fulvic acid models were above 22%, except for the UV spectral range (220–400 nm). The absorbance UV spectra exhibited the highest correlation with the fulvic acids (RMSECV = 49.89 and R^2^ = 0.391). The results of the prediction test revealed a RMSEP of 47.11 (19.78%) and a RPD of 1.61 for the FA.

The results of the humic acids models revealed an improvement in the performance of the PLSR models for higher wavelengths. The best model was for the NIR spectral range (700–1000 nm) with a RMSECV of 82.40 (i.e., 17.07%) and a moderately high Pearson correlation coefficient (R^2^ = 0.538). A RMSEP of 102.35 (i.e., 21.20%) and a RPD of 1.27 were obtained for the independent prediction test. PLSR models for TKN, CN and HI were not very robust. The selected spectral range was 700–1000 nm for both TKN and CN, while the visible range was the best for the humification index. Finally, the regression model for the humification ratio was successful (RPD = 2.23). The best model was for the visible spectral range (400–700 nm) with a high Pearson correlation coefficient (R^2^ = 0.882) and a RMSECV of 1.36 (i.e., 9.25%). A RMSEP of 1.81 (i.e., 16.82%) was obtained for the independent prediction test.

## 4. Discussion

The wastewater biosolid composting process has been associated with the progressive transformation of several organic matter chemical fractions [9,11]. Our results were in accordance with previous research as we obtained significant changes in the organic matter chemical fractions (TEC, HA, FA), humification ratios and other chemical parameters through the compost pile (Table 1). Besides, the temporal evolution of the compost chemical fractions and humification ratios was not associated with a linear trend because their evolution is controlled by the development of several microbial phases (i.e., mesophillic, thermophillic or cooling stages) [1,9]. That complex microbiological and chemical temporal pattern makes the proper identification of the compost maturity difficult. However, the quantification of several composts’ chemical fractions with spectroscopy has been demonstrated as a feasible method for controlling the humification process and compost maturity [12]. Thus, monitoring compost chemical fractions is a suitable way for controlling the composting process.

Most of the previous studies have focused on the spectral measurement of fresh or dried compost samples, the application of standard extraction methods to quantify chemical fractions, and a final statistical analysis to relate the solid compost spectra with the parameters quantified in the liquid compost extracts [24,39,40,41]. These studies employed very diverse spectral acquisition methods. For example, Ilani et al. [24] configured a system to measure bidirectional reflectance of (fresh and dry) compost samples placed a short distance away from light sources and the spectroradiometer (with a spectral range of 350–2500 nm). The performance of the chemical parameters’ regression models was sensitive to the humidity content of the compost samples. Other authors employed a laboratory-based dual-beam spectrophotometer for the absorbance of dried and ground compost samples [39,40,41]. Other studies analyzed the spectra of compost extracts (instead of the solid compost) obtained from an experimental laboratory scale reactor that required two to 10 days to obtain the extracts [42]. These last four cited studies employed Fourier Transform NIR (FT-NIR) spectrometers with a maximum spectral range between 12,000 and 3800 cm^−1^ (i.e., 833–2632 nm).

Our methodological approach is slightly different because we combined a highly efficient one-step compost extraction method for the quantification of chemical fractions [26] and the spectroscopic analysis (in the UV, visible and SW-NIR spectral regions) of the liquid compost extracts themselves. The laboratory procedure for quantifying organic matter chemical fractions implies their extraction from the solid compost with an extracting solution (e.g., sodium pyrophosphate). This extraction procedure is relatively easy, could be developed by the chemical technicians of a typical WWTP laboratory, and does not need a great inversion in laboratory equipment and reactants. In addition, the inherent physical-chemical heterogeneity of solid compost samples is minimized because the extracting solution separates the organic matter chemical fractions from the rest of the solid material. Thus, the liquid compost extracts contain a chemical fraction–rich solution that is very suitable for spectroscopic analysis without the interference of different compost humidity content or the presence of highly heterogeneous particles (in size and spectral response).

This study analyzed absorbance spectra obtained in the UV spectral region with a dual-beam spectrophotometer and transmittance spectra obtained in the VNIR with a modular spectroradiometer associated with an external light source. Wastewater or compost spectra in the VNIR spectral range are frequently characterized by the absence of diagnostic bands for chemometric analysis [43]. On the contrary, the presence of diagnostic bands or differential spectral patterns in the UV is more frequent [21]. PLSR allows a feasible chemometric analysis by taking into account the full spectral range (or spectral regions) instead of single bands. PLSR can be used with a large number of explanatory variables, generally providing regression models with the highest predictive ability with the smallest number of factors as compared with other regression methods such as ordinary least squares estimator or ridge regression [44]. The employment of partial least square regression for compost parameters spectroscopic analysis is well established [23,24,39,40].

The PLSR models’ selection was based on the lowest RMSECV values and the usefulness of the models was based on recommended thresholds for the RPD (Table 2). Effective regression models were reported for the total organic matter (RPD = 2.68), humification ratio (RPD = 2.23), total exchangeable carbon (RPD = 2.07), total organic carbon (RPD = 1.66) and fulvic acids (RPD = 1.61). All these models were obtained with the VNIR spectroradiometer, thus suggesting its utility for the quantification of relevant parameters of the compost maturity degree. The best TOM model was obtained with seven latent variables for the short-wave near-infrared spectral range (700–1000 nm), R^2^ = 0.628 in the cross-validation and a RMSEP of 12.14%. The most suitable HR model was obtained with four latent variables for the visible spectral range (400–700 nm), R^2^ = 0.882 in the cross-validation and a RMSEP of 16.82%. The best TEC model was determined with four latent variables for the visible and short-wave near-infrared spectral range (400–1000 nm), R^2^ = 0.810 in the cross-validation and a RMSEP of 14.14%. PLSR models for TOM, HR and TEC were the most confident, as indicated by the residual predictive deviation (Table 2). We obtained values higher than two for these variables (RPD = 2.68 for TOM, RPD = 2.23 for HR and RPD = 2.07 for TEC), which is a threshold proposed to identify successful models [37].The accuracy of these PLSR models was comparable with previous studies that employed even wider spectral wavelength ranges. Galvez-Sola et al. [41] employed a FT-NIR spectrometer (830–2600 nm) and reported a good model performance for total extractable carbon (RPD = 2.26) and total organic matter (RPD = 2.13) and a less robust regression model for the humification ratio (RPD = 1.76).

The best TOC model was obtained with four latent variables for the short-wave near-infrared spectral range (700–1000 nm), R^2^ = 0.607 in the cross-validation and a RMSEP of 26.60%. The most suitable FA model was determined with three latent variables for the ultraviolet spectral range (220–400 nm), a RMSECV of 20.94% (R^2^ = 0.391) and a RMSEP of 19.78%. The residual predictive deviation of TOC and FA was slightly higher than 1.6 (RPD = 1.61 for FA) which is a value associated with moderately successful models. Finally, the HA prediction model obtained a value slightly lower than 1.4 which is the threshold employed for poorly moderately successful models. Galvez-Sola et al. [41] reported a RPD = 2.73 for TOC, a RPD = 2.12 for FA and a lower value for HA (RPD = 1.55) as well. Vergnoux et al. [39] also reported lower prediction capabilities for HA regression models with spectra acquired with a FT-NIR spectrometer.

The proposed methodology (VNIR spectroscopy + liquid compost extracts) was feasible for the quantification of relevant compost chemical parameters commonly employed for the monitoring of the compost maturity process. This approach was based in the employment of liquid compost extract instead of solid compost samples, resulting in a simpler and less time-consuming sample preparation. In addition, the most suitable sensor (three of four chemical fractions) was a visible and short-wave near-infrared (SW-NIR) spectroradiometer. This kind of sensor is highly modular and customizable, less expensive than laboratory-based FT-NIR spectrophotometers or visible and full NIR spectroradiometers (350–2500 nm), and employs easy-to-replace parts which is important for wide implementation of the methodology at medium-size compost facilities. The spectroradiometer and the illumination source are connected to the cuvette holder by fiber optic wires, which prevent them from getting dirty or damaged from vibrations or daily operation. The sensors are the weakest part of the chain in real-time monitoring and control of sewer systems and wastewater treatment plants [17]. In this sense, the fiber optic sensor (FOS) possesses several advantages over conventional devices, mainly due to the characteristics of the optical fiber itself. This is because FOS can be made very small and thin, resistant to harsh chemical environments (as the conditions in the compost tunnels) and impervious to electromagnetic interference [20]. This combination of a less time-consuming compost sample preparation and a versatile and sufficiently accurate spectroscopy system are promising tools for the development of near-real-time monitoring of the composting process.

## 5. Conclusions

The management of the composting process remains highly un-automatized and requires time-consuming laboratory analyses. In this sense, the implementation of sensors and methodologies capable of a fast and quantitative assessment of the compost maturity status are needed. We proposed a methodological approach focused in the development of an easy-to-implement, efficient and cost-effective protocol for compost maturity assessment and near-real-time monitoring (about 2 h from the compost sampling to the final results). This approach is based on the employment of a VNIR spectroradiometer with a cuvette holder and an external illumination source for spectroscopic analyses of liquid compost extracts. Our previously proposed one-step extraction procedure is feasible, less time-consuming, and the liquid compost extracts were highly suitable for spectroscopy. The employment of those liquid compost extracts allowed the development of successful PLS regression models for the total organic matter, humification ratio, total exchangeable carbon and total organic carbon with the VNIR spectroradiometer. This kind of device is available on the market at a reasonable cost, its operation is affordable and the statistical models employed for the prediction of the compost chemical parameters were successful enough to allow better monitoring of the composting process.

## Figures and Tables

**Table 1:** Summary statistics of compost analytical parameters and humification ratios for all compost samples (*n* = 84). ANOVA results (*p*-value and significance level) are shown in the last column.

Parameter	Minimum	Maximum	Mean	St. Dev.	CV (%)	ANOVA
TOM (%)	66.3	75.4	70.8	3.0	4.2	<0.001 ***
TOC (%)	36.1	41.2	38.6	1.7	4.3	<0.001 ***
TEC (mg/L)	407.3	903.2	653.0	132.4	20.3	<0.001 ***
FA (mg/L)	121.4	359.6	237.5	67.0	28.2	<0.001 ***
HA (mg/L)	144.4	627.2	415.4	123.7	29.8	0.036 *
TKN (%)	2.6	3.6	3.0	0.2	8.4	0.069 ns
C/N	10.9	15.5	12.9	1.1	8.2	0.167 ns
HR (%)	10.0	24.6	17.0	4.0	23.5	<0.001 ***
HI (%)	3.5	17.1	10.8	3.5	32.0	0.012 *

Significance levels: ns: not significant; * *p* < 0.05; *** *p* < 0.001.

**Table 2:** Results of the partial least square regression (PLSR) cross-validation (CV) and prediction (P) models of compost analytical parameters and humification ratios.

Parameter	Spectral Range (nm)	LV	Cross-Validation			Prediction Test		
			R^2^	RMSECV	RMSECV (%)	RMSEP	RMSEP (%)	RPD
TOM	220–400	4	0.476	2.18	23.81	-	-	-
	400–1000	2	0.537	2.06	22.50	-	-	-
	400–700	2	0.548	2.04	22.21	-	-	-
	700–1000	7	0.628	1.82	19.89	1.11	12.14	2.68
TOC	220–400	4	0.476	1.21	23.81	-	-	-
	400–1000	5	0.511	1.168	22.99	-	-	-
	400–700	2	0.548	1.129	22.22	-	-	-
	700–1000	4	0.607	1.05	20.64	0.99	26.60	1.66
TEC	220–400	4	0.400	101.20	22.00	-	-	-
	400–1000	4	0.810	57.08	12.41	65.02	14.14	2.07
	400–700	4	0.808	57.09	12.41	-	-	-
	700–1000	4	0.724	68.74	14.95	-	-	-
FA	220–400	3	0.391	49.89	20.94	47.11	19.78	1.61
	400–1000	3	0.286	54.06	22.70	-	-	-
	400–700	4	0.196	57.31	24.06	-	-	-
	700–1000	3	0.255	55.22	23.18	-	-	-
HA	220–400	4	0.000	123.60	25.60	-	-	-
	400–1000	3	0.463	88.68	18.37	-	-	-
	400–700	5	0.483	86.67	17.95	-	-	-
	700–1000	2	0.538	82.40	17.07	102.35	21.20	1.27
TKN	220–400	2	0.000	0.29	27.58	-	-	-
	400–1000	1	0.000	0.28	26.82	-	-	-
	400–700	1	0.000	0.28	26.92	-	-	-
	700–1000	1	0.000	0.28	26.79	0.25	38.37	0.82
CN	220–400	1	0.000	1.18	26.16	-	-	-
	400–1000	1	0.000	1.11	24.65	-	-	-
	400–700	1	0.000	1.12	24.72	-	-	-
	700–1000	1	0.000	1.11	24.63	0.95	34.71	1.04
HR	220–400	4	0.450	2.94	20.05	-	-	-
	400–1000	4	0.828	1.65	11.23	-	-	-
	400–700	8	0.882	1.36	9.25	1.81	16.82	2.23
	700–1000	4	0.751	1.98	13.51	-	-	-
HI	220–400	2	0.000	3.41	25.05	-	-	-
	400–1000	4	0.527	2.33	17.13	-	-	-
	400–700	5	0.616	2.09	15.39	3.35	32.20	1.10
	700–1000	2	0.585	2.19	16.10	-	-	-
